# Isolation and characterization of cidofovir resistant vaccinia viruses

**DOI:** 10.1186/1743-422X-5-58

**Published:** 2008-05-14

**Authors:** Marie N Becker, Maria Obraztsova, Earl R Kern, Debra C Quenelle, Kathy A Keith, Mark N Prichard, Ming Luo, Richard W Moyer

**Affiliations:** 1 University of Florida, Gainesville, FL, USA; 2 University of Alabama at Birmingham, Birmingham, AL, USA

## Abstract

**Background:**

The emergence of drug resistant viruses, together with the possibility of increased virulence, is an important concern in the development of new antiviral compounds. Cidofovir (CDV) is a phosphonate nucleotide that is approved for use against cytomegalovirus retinitis and for the emergency treatment of smallpox or complications following vaccination. One mode of action for CDV has been demonstrated to be the inhibition of the viral DNA polymerase.

**Results:**

We have isolated several CDV resistant (CDV^R^) vaccinia viruses through a one step process, two of which have unique single mutations within the DNA polymerase. An additional resistant virus isolate provides evidence of a second site mutation within the genome involved in CDV resistance. The CDV^R ^viruses were 3–7 fold more resistant to the drug than the parental viruses. The virulence of the CDV^R ^viruses was tested in mice inoculated intranasally and all were found to be attenuated.

**Conclusion:**

Resistance to CDV in vaccinia virus can be conferred individually by at least two different mutations within the DNA polymerase gene. Additional genes may be involved. This one step approach for isolating resistant viruses without serial passage and in the presence of low doses of drug minimizes unintended secondary mutations and is applicable to other potential antiviral agents.

## Background

Although smallpox was effectively eradicated in the 1970's, a recent concern has been the use of the remaining controlled laboratory stocks or engineered laboratory strains as potential bioterrorist weapons. Furthermore, outbreaks of monkeypox, a virus indigenous to equatorial Africa, have occurred recently in both the US and Western Africa in human populations and demonstrate the potential of viruses to be rapidly transmitted throughout the world [1]. The vaccine for smallpox, vaccinia virus (VV), confers cross protection to other orthopoxviruses including those that infect humans, e.g. monkeypox and cowpox viruses. Although cidofovir (CDV) has been approved under an investigational new drug application for the emergency treatment of certain orthopoxvirus infections, it is not orally bioavailable and is nephrotoxic. Recently a lipophilic derivative of CDV has been shown to have increased bioavailability while retaining effectiveness against orthopoxvirus infections *in vitro *and *in vivo *and is currently in phase I/II clinical studies [2-4].

CDV is a nucleotide analog and thus the proposed target of its interaction is the viral DNA polymerase. CDV resistant (CDV^R^) orthopoxviruses were isolated previously via serial passage [5,6]. Subsequently, in the case of CDV^R ^VV the mutations responsible for resistance were mapped to the viral DNA polymerase [5,7]. The virus described by Andrei et al. contains two mutations within the DNA polymerase and those isolated by Smee et al. contain 5 mutations [5,7,8]. Our goal was to identify additional mutations and through a process that would promote the isolation of resistant viruses containing single mutations and to map those mutations to help provide insight about the interaction of the drug with the enzyme.

## Results

### CDV cytotoxicity, effective concentration for abolishing VV plaque formation

**Figure 1:**
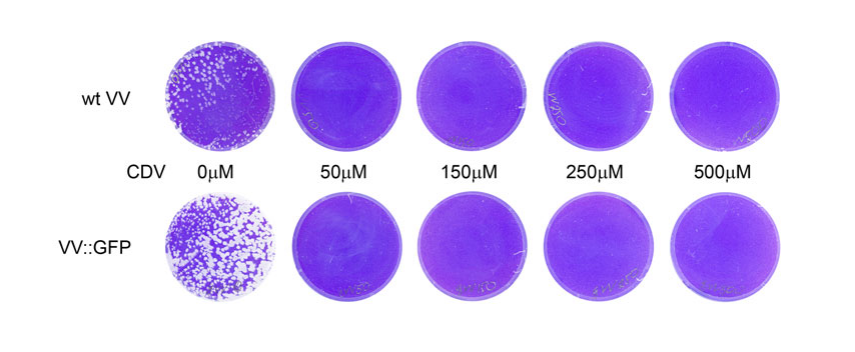
**VV sensitivity to CDV**. Drug concentrations of as low as 50 μM are effective at abolishing plaque formation.

We first established the concentration of CDV that would effectively eliminate wt VV plaques without having significant cytotoxic effects on the BSC40 cells (Figure 1). The concentrations that were utilized were based on previous work [9] which indicated that the EC_50 _for CDV is ~50 μM. Concentrations as low at 50 μM were effective in eliminating plaque formation. We chose to use 150 μM, a value three times the EC_50 _as the concentration for selection of mutants and no cytotoxicity was apparent at this concentration in these cells. Two different virus strains, VV WR and VV TK::GFP were initially used to isolate resistant mutants. The presence of GFP made the identification of small plaques much easier; however, this parental virus is thymidine kinase (TK) negative thus attenuating the virus and rendering it an unsuitable backbone to later assess the CDV^R ^virus phenotype in animals [10]. Indeed, since TK indirectly impacts DNA synthesis, a second goal of these studies was to determine whether the inhibitory concentrations of CDV and subsequent mutant selection were impacted by deletion of this enzyme. This was deemed to not be the case as mutants were readily isolated from either virus at comparable concentrations of CDV and is consistent with results published previously [11]. To insure selection of independent mutations, 10 individual plaque purified stocks from both VV WR and VV TK::GFP, were used as the parental lines for the isolation of resistant mutants. A total of six independent resistant viruses were isolated and the DNA polymerase, E9L, gene was sequenced from each virus (Table 1). Each of these viruses contained a mutation(s) in the viral DNA polymerase.

**Table 1:** CDV resistant VV E9L genotype

Virus	Parental virus strain	Original mutation in E9L	E9L sequence of reconstructed virus
CDV^R ^1	VV WR	A314V	A314V
CDV^R ^2	VV TK::GFP	A314V	A314V
CDV^R ^11	VV TK::GFP, line 11	A314V; P738S	ND
CDV^R ^14	VV WR, line 14	A314V	ND
CDV^R ^15	VV WR, line 15	M671I	M671I, ΔK174
CDV^R ^16	VV WR, line 16	ΔK174	ΔK174

### Marker rescue and mapping of mutations conferring resistance

**Figure 2:**
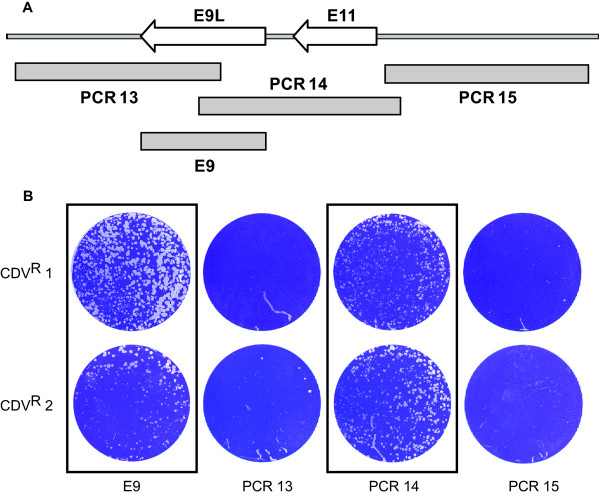
**Mapping and marker rescue of recombinant viruses**. **A**. Map of PCR fragments in the E9L region that were used for marker rescue mapping experiments and reconstruction of resistant viruses. **B**. Results of mapping experiment for CDV^R ^1 and 2. Monolayers of BSC 40 cells infected with virus resulting from infection/transfections of VV WR and the indicated PCR fragments and stained with crystal violet. CDV was present at 150 μM. Only those PCR fragments that contain the mutation conferring resistance to CDV are capable of producing recombinant viruses that were detectable in the plaque assay.

In order to confirm that the mutation detected in the E9L gene was responsible for the CDV resistance, we performed a series of marker rescue experiments and the results of the marker rescue experiments for isolates CDV^R ^1 and 2 are shown in Figure 2. DNA fragments from drug resistant isolates were amplified by PCR and used to transfect wild type VV infected cells (Fig. 2A). Resulting viruses were plaqued in the presence of CDV to score for marker rescue (Fig. 2B). Only PCR products that contained a mutation conferring resistance to CDV should produce plaques, in this example, PCR fragments E9 and 14 (Fig. 2B). To remove the possibility of second site mutations in the original virus, resistant viruses were reconstructed in a wild type VV background by transfecting PCR products containing only a single mutation in E9L. For CDV^R ^1 and 2 we used PCR fragment 14 containing the A314V mutation. For CDV^R^15 a fragment containing the M671I mutation was used and for CDV^R^16, a fragment with the ΔK174 mutation was transfected. The reconstructed viruses are designated with an "A" following the original virus name to distinguish them from the original isolates. Reconstructed viruses containing only the identified mutation in E9L in a wild type VV background were sufficient to confer CDV resistance except for CDV^R ^15. We were unable to reconstruct the CDV^R ^15A virus (Table 1) cleanly despite several attempts and the resulting reconstructed virus always contained two mutations within the E9L gene, the original mutation (M671I) and a second mutation that corresponds to the same mutation found in CDV^R ^16 (ΔK174). This was observed multiple times and indicates quite clearly, that the resistance that allowed the isolation of CDV^R^15 containing the M671I mutation depends on a second contributing mutation in the original CDV^R ^15 isolate outside of the DNA polymerase for resistance. Furthermore, this second site mutation in CDV^R^15, outside the DNA polymerase can be compensated for by a specific second mutation in the DNA polymerase, ΔK174. In contrast, CDV^R ^16A was successfully reconstructed to contain only the original ΔK174 mutation.

### Growth properties of CDV^R ^viruses

**Figure 3:**
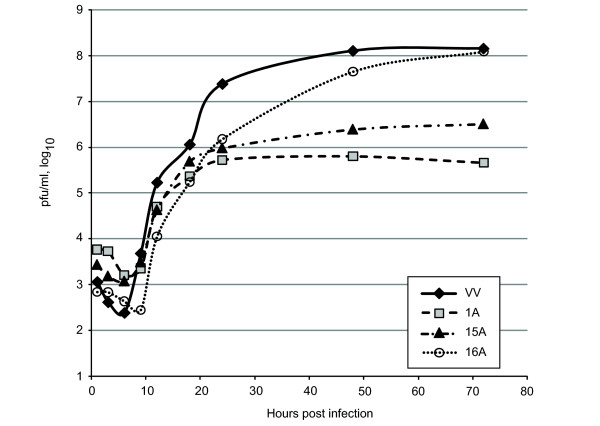
**Growth properties of CDV^R ^viruses**. BSC40 cells were infected with either VV WR; CDV^R ^1A; CDV^R ^15A or CDV^R ^16A at an MOI = 0.02. Samples were harvested at 1, 3, 6, 9, 12, 24, 48, and 72hpi. Samples were titered on CV1 cells and the results graphed.

Each of the three reconstructed viruses, CDV^R ^1A, 15A and 16A were analyzed for their growth properties compared to wt VV. The growth curves in Figure 3 indicate that all three CDV resistant strains grew less well than wild type virus although CDV^R ^16A did produce titers reaching wild type levels after an initial lag in growth. All three resistant strains produced very small plaques compared to wild type virus, with CDV^R ^1A producing "pinpoint" plaques after three days. CDV^R ^1A and 15A ultimately produced much less total virus than wild type or CDV^R^16A.

### Levels of resistance

We confirmed that the viruses were resistant to CDV in two additional cell lines at another laboratory (Table 2). The EC_50 _for CDV was obtained in HFF and Vero cells and compared to two independently obtained strains of parental VV WR. The resistant viruses had EC_50 _values that were 3 to 7 fold higher than the parental virus strains. The greatest resistance was with CDV^R^1A containing the mutation at A314V which produced the smallest plaques and lowest titers.

**Table 2:** Activity of CDV Against Wild Type and CDV Resistant VV using a Plaque Reduction Assay in Human Foreskin Fibroblast and Vero Cells

Virus	HFF EC_50 _(μM)^a^	Vero EC_50 _(μM)^a^	Fold resistance over parental strain (HFF)	Fold resistance over parental strain (Vero)
VV-WR, UAB	28 ± 4.4	62 ± 12	-	-
VV-WR, Moyer	18 ± 9.2	54 ± 2.9	-	-
CDV^R ^1A	122 ± 69	>317 ± 0	7	>6
CDV^R ^15A	98 ± 55	214 ± 17	5	4
CDV^R ^16A	49 ± 4.5	199 ± 2.8	3	4

^a^Values are the mean ± standard deviation of two or more assays.

### Virulence of CDV^R ^viruses in mice

It has been previously reported that CDV^R ^VV is attenuated in mice. We assessed the virulence of our reconstructed viruses in mice inoculated intranasally (Tables 3 and 4) and confirmed that all of our resistant strains were significantly attenuated in this model. No mice were killed by the CDV^R^15A strain even at the highest dose given, so an LD_90 _could not be established.

**Table 3:** Mortality of BALB/c Mice Inoculated Intranasally with Wild Type or CDV Resistant Vaccinia Viruses

	Mortality			
Virus^a^	Number	Percent	MDD^b^	LD_90_
**VV-WR, UAB**^**c**^				
2.8 × 10^4^	10/10	100	7.1	2.9 × 10^3^
2.8 × 10^3^	7/10	70	8.1	--
2.8 × 10^2^	2/10	20	9.0	--
28	0/10	0	--	--
2.8	0/10	0	--	--
				
**VV-WR, Moyer**^**c**^				
1.3 × 10^4^	10/10	100	7.8	<1.3 × 10^4^
1.3 × 10^3^	0/10	0	--	--
1.3 × 10^2^	0/10	0	--	--
13	0/10	0	--	--
1.3	0/10	0	--	--
				
**CDV**^**R**^**1A**^**c**^				
Stock 1.2 × 10^4^	0/10	0	--	>1.2 × 10^4^
1.2 × 10^3^	0/10	0	--	--
1.2 × 10^2^	0/10	0	--	--
12	0/10	0	--	--
1.2	0/10	0	--	--

a. Virus was delivered i.n. in 0.04 (0.02 ml/nostril) ml doses.

b. MDD = Mean Day of Death.

c. Inoculum, PFU/mouse.

**Table 4:** Mortality of BALB/c Mice Inoculated Intranasally with Wild Type or CDV Resistant Vaccinia Viruses

	Mortality			
Virus^a^	Number	Percent	MDD^b^	LD_90_
**VV-WR**^**c**^				
1.6 × 10^4^	15/15	100	8.5	4.4 × 10^3^
1.6 × 10^3^	2/15	13	8.5	--
1.6 × 10^2^	0/15	0	--	--
16	0/15	0	--	--
1.6	0/15	0	--	--
				
**CDV**^**R**^**15A**^**c**^				
Stock, 6 × 10^4^	0/15	0	--	--
6 × 10^3^	0/15	0	--	--
6 × 10^2^	0/15	0	--	--
60	0/15	0	--	--
6	0/15	0	--	--
				
**CDV**^**R**^**16A**^**c**^				
Stock, 8 × 10^5^	3/15	20	8.0	>8 × 10^5^
8 × 10^3^	0/15	0	--	--
8 × 10^2^	0/15	0	--	--
80	0/15	0	--	--
8	0/15	0	--	--

a. Virus was delivered i.n. in 0.04 (0.02 ml/nostril) ml doses.

b. MDD = Mean Day of Death.

c. Inoculum, PFU/mouse.

### Modeling of E9 and location of mutations

**Figure 4:**
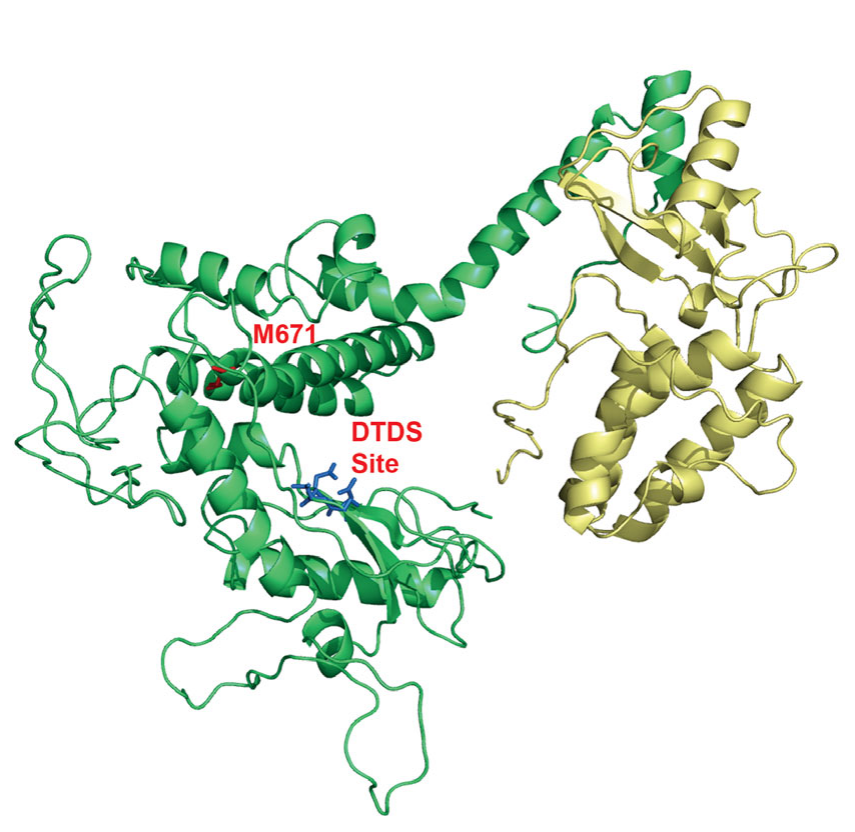
**Model of the catalytic core of E9 polymerase**. The green ribbons correspond to the homologous region as modeled. Met671 is shown as a red stick model and the active site residues Asp, Thr, Asp, Ser are shown as blue stick models. The yellow ribbons are included to show the remaining part of the catalytic domains of the 1TGO polymerase, but there is no significant amino acid sequence homology between the two polymerases. The figure was prepared with PyMol.

**Figure 5:**
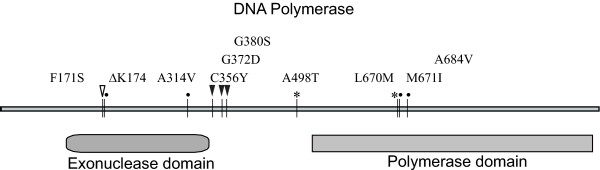
**Domains of E9 and locations of mutations responsible for drug resistance**. The location of the putative exonuclease and polymerase domains is indicated. Mutations conferring resistance to CDV are indicated by circles. In addition to the location of known CDV mutations (circles), mutations conferring resistance to phosphonoacetic acid (closed triangles), cytosine arabinoside (open triangle) and aphidicolin (asterisk) are shown [17–19].

No crystal structures of VV E9 exist in the protein database. However, E9 exhibits significant homology to the type B family of DNA polymerases. Residues 429–809 of E9 could be aligned with residues 279–597 of Thermostable B Type DNA Polymerase from *Thermococcus gorgonariu *with 41% homology (E value = 1e-15). This region represents the catalytic core of the DNA polymerase. By modeling E9 on the crystal structure of *Thermococcus gorgonariu *DNA polymerase (PDB code 1TGO) it appears that the location of the M671I mutation is not far from the active site in the putative polymerase domain (Figure 4). A summary of known mutations conferring drug resistance is presented in Figure 5. A number of mutations cluster in the exonuclease domain, including those at residue 314 as previously noted [5].

## Discussion

Although mutations in DNA polymerase that confer resistance to CDV have been previously isolated, this is the first report of a selection procedure that is sufficient to isolate single mutations conferring resistance. In this study we report that the A314V mutation alone confers significant resistance to CDV. This mutation was isolated several times independently. Previous studies by Andrei et al. (2006) had demonstrated that a mutation of alanine 314 to threonine conferred resistance to CDV; however, higher levels of resistance were obtained when this mutation was in combination with a second mutation, A684V, found in the original isolate [5]. This study used drug concentrations twice as high as our study for the characterization of these viruses. Our lower drug concentrations indicate that even at low doses of drug the development of resistant viral strains can pose a problem.

Two other mutations conferring resistance were also isolated. One is a novel mutation of the deletion of amino acid K174 within the putative exonuclease domain of the DNA polymerase. Again, this mutation alone conferred resistance; however, the level of resistance is not as great as that for A314V. Examination of the CDV^R ^15 mutant provided some of the most interesting results. In our original isolation of CDV^R^15 we found only a single mutation within the E9 gene, however, upon reconstruction this mutation alone cannot confer resistance and attempts to reconstruct this virus resistant to CDV always contained the ΔK174 mutation as well. This implied that the original CDV^R^15 virus we isolated must contain a second mutation elsewhere in the genome other than in the DNA polymerase. Further analysis of CDV^R^15 by marker rescue experiments should allow identification of this second target gene. The lower titers of the CDV^R ^stocks that were produced severely limited the amount of virus that could be used in this model. However, as expected from previous work, all three of our reconstructed virus strains grew somewhat less well than wild type virus and were attenuated in the mice by more than one log [5].

## Conclusion

The results obtained from these studies provides further evidence that the primary but not sole target of CDV is the viral DNA polymerase and that drug resistance can be a significant problem even in the presence of relatively low doses of drug. It is important to note that these drug resistant mutants all had reduced virulence in mice and suggest that the development of these mutants may not contribute to enhanced disease.

### Methods

#### Cells and viruses

Monolayer cultures of BSC40 cells (Dr. Richard Condit) were maintained in Dulbecco's modified Eagle medium (DMEM) supplemented with 10% fetal bovine serum (FBS) (Gibco), 50 IU of penicillin, and 50 μg of streptomycin per ml (Cellgro, Herndon, Va.) [12]. CV1 cells (ATCC, CCL-70) were maintained in minimal essential media (MEM) with Earle's salts supplemented with 5% FBS, 340 mM sodium pyruvate, 50 U/ml penicillin, 50 μg/ml streptomycin and non-essential amino acids. Vero Cells were obtained from ATCC and were maintained in MEM with Earl's salts and the addition of 10% FBS and standard concentrations of L-glutamine, penicillin and gentamicin. Methods for obtaining and passaging human foreskin fibroblast (HFF) cells were described previously [13]. All cell lines were maintained at 37°C in the presence of 5% CO_2_.

The parental viruses used for isolation of CDV^R ^mutants were VV WR and VV TK::GFP. VV TK::GFP contains the GFP gene driven by the synthetic VV early-late promoter inserted into the *thymidine kinase *(*tk*) gene. This virus was generated via standard methods using a pSC65GFP clone in order to recombine the GFP gene into the TK locus of wild type VV [14]. Virus titers were determined by standard plaque assays. To obtain CDV^R ^mutants, 20 independent virus stocks were generated from single plaques from the original VV TK::GFP (lines 1–10) and VV WR (lines 11–20) virus stocks.

#### Cidofovir

CDV was provided by Gilead Sciences, Foster City, CA. Stock solutions of CDV (5 mM) in DMEM without serum was stored at 4°C and protected from light.

#### Isolating independent CDV^R ^mutant viruses

Confluent monolayers of BSC40 cells in 6-well plates were infected with 2 × 10^4 ^PFU/well of either VV WR or VV TK::GFP in DMEM with no supplements except for 150 μM CDV. After 60 min of adsorption an additional 1.5 ml of DMEM with 10% FBS, antibiotics and 150 μM CDV was added to each well. Plates were incubated at 37°C for 48 h and examined for plaques under the light microscope, or with fluorescence for GFP containing plaques. To isolate identified plaques, the liquid medium was carefully removed and the plaque was scraped with a 1 ml large bore pipette tip and transferred into 1 ml of DMEM without serum and stored at -80°C. Routinely, 2 plaques from each individual virus stock were isolated. The virus from the original plaque was plaque purified one additional time under agarose and in the presence of 150 μM CDV to ensure that it was a single isolate. From these dishes, plaques were picked and subsequently amplified.

#### Sequencing Analysis

DNA sequences of the DNA polymerase (E9L) gene from the viruses were obtained by direct sequencing of the PCR products amplified from the total DNA of infected cells. The DNA was prepared from virus infected cells with the DNeasy Tissue Kit (Qiagen Inc., Valencia,, CA), according to the manufacturer's protocol. The entire E9L gene was PCR amplified using two primers IDT327, 5'-ATGGATGTTCGGTGCATTAATTGGT-3' and IDT328, 5'-TTATGCTTCGTAAAATGTAGGTTTTGAACC-3' and then sequenced with primers that hybridize within E9L to give overlapping sequence data. Sequencing was performed by the University of Florida ICBR DNA Sequencing Core Laboratory.

#### Reconstruction of CDV^R ^mutants by marker rescue

Mapping of the individual mutations conferring resistance and the reconstruction of the mutation(s) in a wild type VV background were performed as described previously [15]. Confluent monolayers of BSC40 cells in 6 well dishes were infected with 5 × 10^3 ^PFU of wt VV WR in a volume of 0.5 ml and 30 min later transfected with 1.5 – 2 μg DNA complexed with 12 μl Lipofectamine 2000 per manufacturer's instructions (Invitrogen). Different PCR products from the mutant CDV^R ^viruses were used for transfection including products containing the entire E9L gene or products containing only portions of E9L gene. A map of the fragments used is found in Figure 2. PCR fragment 13 is approximately 5 kb and contains approximately half of E9L at the 3' end as well as DNA downstream of E9L into E6 (primers 5'-TACGATGTTGTAAAGTGTACGAAGCG-3'; 5'-AGTTAGAGAAATGACGTTCATCGGTG-3'). The 5' portion of E9L is contained in a 5 kb fragment, #14, generated with 5'-TTTGTTTTGGAGCAAATACCTTACCG-3' and 5'-CGAGAGTGGTTGAATGTTTGACTGTG-3'. As a negative control, fragment 15 approximately 2.9 kb upstream of E9L translational start site was used in transfections (5'-AAATAGTCACGCAATTCATTTTCGGG-3'; 5'-TGCTTTTGATGGTAATTTCTGGTGCC-3'). All primers and fragment numbering is from Luttge and Moyer, 2005 The cells were incubated at 37°C for 1 h while rocking, then an additional 2 h without rocking. DMEM containing 150 μM CDV was added to each well. Plates were incubated at 37°C for 48 hr, and then harvested. The resulting viruses were grown on BSC40 cells in the presence of 150 μM CDV. When mapping mutations, the dishes were stained with crystal violet. For reconstruction CDV^R ^plaques were picked from the plaques obtained from the infection/transfection mixture and later amplified on BSC40 cells for stocks. The E9L gene of the reconstructed viruses was sequenced as described above and compared to the original mutation from which it was derived.

#### Drug sensitivity and EC_50 _determination

BSC40 cell monolayers in 60 mm dishes were infected with 200 PFU of VV WR, VV TK::GFP or CDV^R ^mutant virus suspension in 0.5 ml DMEM without serum and with CDV concentrations of 0, 50, 150, 250 or 500 μM. After adsorption for 60 min at 37°C, the medium was carefully aspirated and the wells were overlaid with 1% agarose mixed with an equal volume of 2 × DMEM, 10% FBS and the same concentration of CDV used during the initial infection. The plates were incubated for 4 days at 37°C and then stained with 0.26% crystal violet in 10% ethanol, 22% formaldehyde and plaques were counted.

#### VV plaque reduction assays

HFF cells were added to 6-well plates two days prior to the assay. On the day of assay, drug at two times the final desired concentration was diluted serially 1:5 in 2× MEM with 10% FBS to provide six concentrations. Culture medium was aspirated from triplicate wells for each drug concentration and 0.2 ml per well of diluted virus was added which yielded 20–30 plaques per well. The plates were incubated for one h with shaking every 15 minutes. Equal volumes of 1% agarose and drug solutions were mixed and added to each well in 2 ml volumes and the plates incubated for three days. Cell monolayers were stained with neutral red and plaques were enumerated using a stereomicroscope at 10× magnification. 50% effective concentration (EC50) values were calculated by standard methods.

#### Growth Curves

Growth properties of the reconstructed CDV^R ^viruses were compared to growth of wild type VV. BSC40 cells (1 × 10^5^) in twelve well dishes were infected individually with each virus, wt VV, CDV^R ^1A, CDV^R ^15A, CDV^R ^16A, at an MOI = 0.01 PFU/cell, in duplicate. After 1 h adsorption, the virus was removed and the cells washed with PBS. One ml of DMEM containing 10% FBS was added to cells. Infected cells were incubated at 37°C and harvested by scraping at the following time points: 1, 3, 6, 9, 12, 18, 24, 48, 72 h post infection. The virus was released from the cells by 3 freeze thaw cycles and titered on CV1 cells.

#### Virulence in Mice

Female BALB/c mice, 3 weeks of age, were obtained from Charles River Laboratories, Raleigh, North Carolina. Mice were group housed in microisolator cages and utilized at a quantity of 10–15 mice per group. Mice were obtained, housed, utilized and euthanized according to USDA and AAALAC regulatory policies. All animal procedures were approved by University of Alabama at Birmingham, Institutional Animal Care and Use Committee prior to initiation of studies. BALB/c mice were anesthetized with ketamine-xylazine prior to virus inoculation. VV infections were initiated by intranasal inoculation of media containing varying concentrations of wild type and drug resistant mutants of VV ranging from 8 × 10^5 ^to approximately 1 PFU/animal, depending on the titer of each virus stock. Virus suspension was instilled into both nostrils using a micropipetor and a total volume of 40 μl per animal. For these experiments mice were checked for mortality at least once daily for 21 days, but twice daily during the period when peak mortality was expected to occur. The mortality observed for the wild type virus, such as VV WR, was compared with that observed with the CDV^R ^VV.

#### Modeling

The amino acid sequence of E9 was aligned with that of Thermostable B Type DNA Polymerase from *Thermococcus gorgonariu *(derived from PDB file 1TGO) by Blast. A homologous model was calculated based on the amino acid sequence alignment and the known structure 1TGO using Modeller (version 9.2) [16]. The model structure was displayed by PyMol (Delano Scientific, San Carlos, CA).

## List of abbreviations

Wild type: wt; effective concentration: EC_50;_ vaccinia virus Western Reserve strain: VV WR; lethal dose 90%: LD_90;_ Dulbecco's modified Eagle's medium: DMEM; hours post infection: hpi; plaque forming unit: PFU.

## Competing interests

The authors declare that they have no competing interests.

## Authors' contributions

MNB contributed to the experimental design, sequence alignments, data analysis, and drafted the manuscript. MO isolated the resistant viruses and mapped the mutations. ERK contributed to the experimental design and provided a critical review of the manuscript. DCQ directed all mouse experiments and analyzed the resulting data. KAK contributed to the acquisition and interpretation of data. MNP contributed to the interpretation of data and the critical review of the manuscript. ML modelled the DNA polymerase. RWM contributed to the experimental design and assisted in writing the manuscript.
